# Visual Working Memory Capacity and Proactive Interference

**DOI:** 10.1371/journal.pone.0002716

**Published:** 2008-07-23

**Authors:** Joshua K. Hartshorne

**Affiliations:** Department of Psychology, Harvard University, Cambridge, Massachusetts, United States of America; Victoria University of Wellington, New Zealand

## Abstract

**Background:**

Visual working memory capacity is extremely limited and appears to be relatively immune to practice effects or the use of explicit strategies. The recent discovery that visual working memory tasks, like verbal working memory tasks, are subject to proactive interference, coupled with the fact that typical visual working memory tasks are particularly conducive to proactive interference, suggests that visual working memory capacity may be systematically under-estimated.

**Methodology/Principal Findings:**

Working memory capacity was probed behaviorally in adult humans both in laboratory settings and via the Internet. Several experiments show that although the effect of proactive interference on visual working memory is significant and can last over several trials, it only changes the capacity estimate by about 15%.

**Conclusions/Significance:**

This study further confirms the sharp limitations on visual working memory capacity, both in absolute terms and relative to verbal working memory. It is suggested that future research take these limitations into account in understanding differences across a variety of tasks between human adults, prelinguistic infants and nonlinguistic animals.

## Introduction

Visual working memory (WM) capacity–the amount of visual information that can be stored over brief delays–is decidedly underwhelming. Present an observer with two human faces to remember over a brief delay. Then present the observer again with two faces and ask if they both match the faces in the memory set. Humans are well below ceiling on even this very minimal task [1]. A small but intense debate has erupted in recent years over whether humans can remember as many as four different attributes (e.g., color, orientation, etc.) of four line segments over a brief delay [2], [3], [4], [5]. This obscures the fact that four attributes of four line segments is not much.

These sharp limitations in WM for visual information are particularly striking when surveying the literature on WM for verbal information. Historically, very careful experimental manipulations have been required to demonstrate that the capacity of WM is *only* four chunks of verbal material and not more [6], [7], in stark contrast with the visual literature, where careful manipulations are necessary to show that capacity is *as many* as four chunks [2], [3], [4], [5], [8].

With training and the use of simple strategies, humans can be taught to maintain lists of 80 to 90 words in WM [9], [10]. I am unaware of any studies of visual WM that reach even that order of magnitude. Alvarez and Cavanagh suggest a theoretical upper limit of five to six visual objects of minimal complexity, with the number of objects decreasing as complexity increases [2]. Moreover, training studies typically report moderate improvements in visual WM capacity at best, with a number of studies showing no improvement [1], [11], [12], [13], [14], [15].

In one particularly relevant study, Zhang and Simon [16] found that literate Chinese observers could recall three times as many easy-to-name Chinese characters than difficult-to-name Chinese radicals (components of characters), even though the characters actually included the tested radicals, making the former considerably more visually complex. Though this study was not without confounds [6], it is nonetheless striking. It moreover seems telling that, in order to get a pure assessment of visual WM capacity, researchers routinely employ strategies to prevent participants from recoding the stimuli verbally and thus improving performance [4], [17]. I am not aware of a similar concern among researchers that observers in verbal WM tasks may be using a visual strategy.

Thus, it appears that visual WM capacity is quite limited, both in absolute terms, and in comparison with verbal WM. However, it remains to be shown that this capacity difference is not illusory. The tasks used to probe verbal and visual WM capacity are typically quite different (the Zhang and Simon study being a notable exception). Verbal WM capacity is typically assessed by having the observers reproduce the memory items orally or through writing, whereas visual WM tasks typically involve accepting or rejecting a test item as being a member of the memory set (see below for further discussion of the implications). Also, while the ability to maintain 4 extremely simple visual objects appears to pale in comparison to maintaining 90 words, it may not be appropriate to equate words and visual objects–either on the representational level (i.e., which one requires more computational and representational resources to maintain) or on the level of actionable information (i.e., which one is more *useful* to maintain in WM).

Fully understanding the exact limits of visual WM capacity is a non-trivial task. In this paper, I will address one particular concern. Before introducing this study, I wish to give at least one reason that WM capacity matters in addition to familiar questions of understanding the nature of computation and the architecture of the mind [6], [7]. It has been noted [18] that language appears to play an indirect role in a number of the differences between linguistically competent adults on the one hand and pre-linguistic infants or non-linguistic primates on the other. For instance, only humans who have learned number words can represent large numbers (e.g., 20, 200, or 2,000) exactly [19], [20], [21]. Similarly, linguistically competent humans tend to pass standard false belief tasks [22], while infants and non-human primates may not, and language learning seems to directly predict this developmental change in humans [23]. The use of certain navigational cues (e.g., *left of the blue wall*) in reorientation tasks seems to be likewise restricted to linguistically competent humans, and it can be eliminated by interfering with linguistic processing [18].

It has been claimed that the role of language in successful performance of these and other tasks is to allow for the creation and representation of new concepts crucial to the task [18]. In contrast, I note that all these tasks have clear WM components. It may well be that the role of language in these tasks is to increase WM capacity, thus allowing participants to maintain crucial information over a delay. Therefore, I believe that fully understanding WM capacity for different modalities holds important theoretical implications even beyond the crucial questions of mental architecture.

In the present study, I investigated whether the rather poor performance of visual WM typically reported is due to an outsized effect of proactive interference. Proactive interference (PI) occurs when processing on one trial negatively affects performance on a subsequent trial [24], [25]. In verbal tasks, it appears to be a major cause of forgetting [24]; [see also 26]. Importantly, PI appears to be a major factor in individual differences in verbal WM capacity [27]. PI is particularly likely to happen when the test probe matches an item from a previous memory set (“item-specific proactive interference”), or when different stimuli appear on each trial but are members of the same semantic category such as place names (“item-nonspecific proactive interference”) [28], [29]. The relationship between the two types of PI is not clear, but both appear to rely on a common neural substrate [28].

There are two reasons PI might lead to low visual WM capacity estimates, both in absolute terms and relative to verbal WM. First, the paradigmatic verbal WM task requires only a dozen or so trials in order to establish capacity [30], [31]. This is because the tasks use whole-report: the participants repeat all the words from the memory set. In contrast, visual WM tasks involve accepting or rejecting a probe, in which case chance accuracy is 50%. Researchers typically estimate capacity using a formula that considers hits, correct rejections, and the number of stimuli in the memory set [13], [32]. Thus, many more trials are needed–frequently hundreds–and each probe may be shown dozens of times [3], [5], [8], [33]. It is not known, even in the verbal domain, how PI accumulates over hundreds of trials and dozens of presentations of the same stimuli. If the ceiling for PI accumulation is high, poor performance on visual WM tasks could be due to massive PI of a degree simply not seen in verbal tasks.

Furthermore, it is possible that visual WM is particularly susceptible to PI. Although some researchers have suggested that PI may be a result of domain-general processes, neuroimaging results are mixed [28], [34], [35].

Directly comparing verbal and visual PI in a quantified way is difficult, as pointed out above, because matching stimuli across modalities is a non-trivial task, and it is in any case beyond the scope of the present study. In this study, I investigate to what degree PI depresses estimates of visual WM capacity. Furthermore, as PI has only been very recently described in visual WM [17], [28], [34], [35], the experiments in this study also serve to more thoroughly probe the nature of PI in visual WM tasks and consider whether it shows the same behavioral signatures as verbal PI.

### Overview of Experiments and Results

#### Experiment 1

Experiment 1 used a modified recent probes paradigm, which was introduced in the verbal domain by Monsell [36] and which has been used to show relatively small decrements in visual WM performance due to item-specific PI [17], [29], [34], [35].

In the standard version, several visual stimuli are displayed. After a brief retention period, a probe is presented and the observer responds as to whether the probe matches any of the items in the memory set. The crucial manipulation is that in one condition, the non-match probe matches a memory item on the previous trial. In the other condition, the non-match probe is not novel–constraints on the number of available stimuli prevent this ideal manipulation–but at least has not been used in the current or previous trial. Accuracy in the “recent probe” condition is typically a few percentage points lower than in the “non-recent probe” condition, which is instructive, but cannot possibly account for low visual WM capacity estimates by itself.

These experiments were limited in that while the “non-recent probe” did not appear on trial N-1 (the previous trial), it could–and, in some experiments, almost certainly did–appear on trial N-2 (the trial before last). Thus, the “non-recent probe” condition itself may suffer from PI. (Given that all stimuli came from the same semantic category, such as “faces,” the “non-recent probe” condition certainly suffered from item-nonspecific PI, something that is not addressed in the present experiment, but is in subsequent experiments.)

Experiment 1 used a modified recent probes paradigm, where the “recent probe” on trial N could have most recently appeared in trial N-1, N-2, N-3, N-4, N-5 or N-8. Again, there is no perfect baseline with a completely novel probe, but this experiment will at least give us a better sense of how stimulus-specific PI decays over time.

In Experiment 1, I find that item-specific PI does last across 3–4 trials of an experiment and thus may be a larger factor than previous experiments have suggested. However, it appears to decay entirely within 4–5 trials, a fact which sets an upper bound on the influence of item-specific PI.

#### Experiment 2

Experiment 2 estimates the combined effect of item-specific and item-nonspecific PI on measured visual WM capacity. Ten highly dissimilar but non-nameable stimulus sets were assembled. Participants performed a change-detection task similar to that in the previous experiment. Every 10 trials the stimulus set switched. This way, the first trial with each stimulus set (low item-specific and item-nonspecific PI condition) could be contrasted with the 10^th^ trial using that stimulus set (high item-specific and item-nonspecific PI condition). Importantly, Cowan's K (an estimate of capacity; see below) can be computed in both the high- and low-PI conditions and directly compared.

Experiment 2a consisted of 6 blocks of 10 sets of the 10-trial mini-blocks described above. In Experiment 2b, the order of the trials was randomized for 3 of the 6 blocks, thus allowing a direct, within-subjects comparison between the high-PI condition, the low-PI condition, and a typical WM capacity experiment.

In Experiment 2, I find that measured visual WM capacity declines 17% from the low-PI to the high-PI condition, with the “typical” condition producing results intermediate between the two.

#### Experiment 3

Changing stimulus sets every 10 trials in Experiment 2 should have led to reduced PI on the first trial of each mini-block. The stimulus switch may also capture the participants' attention, causing them to pay more attention to the first trial of each mini-block. Thus, improved performance on that trial may be due to attention, not a reduction in PI. This is a potential concern for all previous studies of item-nonspecific PI, but it has not been explored.

Experiments 3a and 3b test this possibility. In Experiment 3a, the stimuli changed color, but not shape, every 10 trials. In Experiment 3b, participants were given a break after every 10 trials.

Neither experiment found any effect of the attentional manipulations on measured visual WM capacity, suggesting that the decline in measured WM capacity in Experiment 2 is indeed due to the effects of PI.

#### Experiment 4

Experiment 2 estimates the effect of PI on measured visual WM capacity, but does not use a truly PI-free baseline. This is not a failing unique to this study; it is true of every study of PI with visual material of which I am aware, and true of many of the studies involving verbal material as well.

One possibility is to create a very large set of stimuli and only use each stimulus once. This turns out to be impractical. If the probe is sufficiently unlike any of the memory items (e.g., memory items were letters, probe is a picture of an elephant), the task is trivially easy. However, if the probes are too similar to the memory items, discriminability becomes a problem [3]. Creating a sufficiently large set of stimuli that are distinct enough yet not too distinct may be an insurmountable problem, given the number of trials required of each participant.

An alternate possibility is to recruit a very, very large number of participants and test each on only a few trials. The first trial would then be as close to PI-free as can be achieved. To have as much data, measuring only the first trial per participant, as was collected by averaging across trials in Experiment 2 requires 3,050 participants. Recruiting 3,050 participants in the lab is of course impractical. However, it can be done over the Web.

Web-based experiments have been used for well over a decade and are rapidly gaining acceptance. A recent review found that 21% of APA journals have published at least one paper relying on Web-based research [37]. A couple studies have found that Web-based experimental results agree well with laboratory-based results [38], [39].

However, most published Web-based experiments have been questionnaires. Vision experiments typically involve careful controls of display size, timing and other factors that cannot be carefully controlled in a Web-based paradigm. That said, such factors seem less important for the study at hand, though they may increase the variability in the data. Thus, this experiment in some aspects was also a trial run for vision and memory experiments on the Internet.

In Experiment 4, I test 3,000+ participants in a visual WM task in order to establish as close as possible a PI-free baseline (the first trial). The results replicate Experiments 1 and 2, suggesting that visual PI reaches asymptote within several trials, and while it has a respectable and statistically significant effect on measured visual WM capacity, the effect is not large enough (∼12%) to explain away the typical low estimates of visual WM capacity.

## Methods

### Experiment 1: Method

#### Participants

Participants in all experiments were volunteers from Harvard University and the surrounding community, who were compensated either with payment or course credit. All human subject involvement was governed by the policies of Harvard University Committee on the Use of Human Subjects in Research. In Experiments 1–3, participants read a description of the experiment and gave written, signed consent. In Experiment 4, the fully anonymous participants read a description of the experiment and clicked a Web link to indicate consent. All participants reported normal or corrected-to-normal visual acuity and normal color vision. A total of 20 participants completed Experiment 1.

#### Equipment

Participants in Experiments 1–3 were tested individually in a normally lit interior room. They sat unrestricted at about 57 cm away from a computer monitor. The experiments were programmed with Psychophysics Toolbox implemented in MATLAB [40], [41].

#### Materials

Three sets of six stimuli were used: non-nameable novel shapes, novel polygons [42], and blue fribbles from different families (used with permission, Michael J. Tarr, http://www.tarrlab.org). Shapes ranged from approximately 2° to 3.2° in diameter and were displayed on a neutral gray background. Stimuli from all experiments in this study can be found in Appendix S1.

#### Procedure

**Figure 1:**
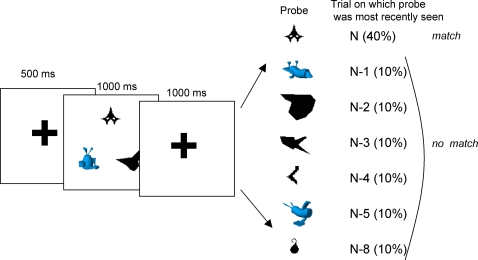
Design of Experiment 1.

The procedure for Experiment 1 is shown in Figure 1. Each trial began with 500 ms of fixation on a red crosshair. Then three stimuli, one from each stimulus group, were displayed equidistantly on an imaginary circle (radius = 2.4°). This memory set was displayed for 1000 ms. After a further 1000 ms of fixation, a single test item was presented at the center of the screen until a response was made. Participants were asked to press one of two keys to report whether the test item matched one of the memory items.

Each participant completed 700 experimental trials divided into 7 blocks. Within a block, stimuli from each stimulus group always appeared in the same location, though these locations switched randomly between blocks. On each block, for 40 of the trials (40%) the probe matched one of the memory set items. On the other 60 trials (60%), the mismatch probes had been most recently used as a memory item on trial N-1, N-2, N-3, N-4, N-5 or N-8. In all cases, the test item had not been used in the meantime as a test item. On each block, each mismatch condition occurred between 11 and 13 times (∼10%). The N-8 condition serves as an imperfect baseline.

In order to train participants on the task without exposing them to the stimuli, practice trials involved stimuli highly dissimilar to those used in the experiment. Participants received feedback on incorrect responses only. To minimize verbal naming, participants were required to rehearse a one-syllable word (specified at the beginning of each block) as quickly as they could throughout the block.

### Experiment 2: Method

#### Participants

Twenty participants completed Experiment 2a. Twenty-one participants completed Experiment 2b.

#### Materials

Ten stimulus groups of six objects each were created. Three (non-nameable shapes, polygons and fribbles) were used in Experiment 1. The others were faces, oriented lines, rotated cubes, colored squares, line-drawings of houses, Sanskrit letters, and rotated 2s, 5s, and 10s. Shapes ranged from approximately 2° to 3.2° in diameter. The stimuli can be found in Appendix S1.

#### Experiment 2a

The purpose of this experiment was to compare visual WM capacity measures under low- and high-PI. Each trial began with 500 ms of fixation on a red crosshair. Then, four stimuli from a single stimulus group (e.g., faces) were displayed around an imaginary circle (radius = 3°) for 1000 ms. The memory set was followed by a 1000 ms retention interval during which participants fixated on the crosshair. Then, a test item was presented. To minimize extraneous search and comparison factors, the test item was presented in the location of one of the memory set items. Participants were to respond as to whether it matched the memory item that had been in that location. The test item matched in 50% of trials. When it did not match (50%), it was not the same as *any* of the memory items for that trial.

**Figure 2:**
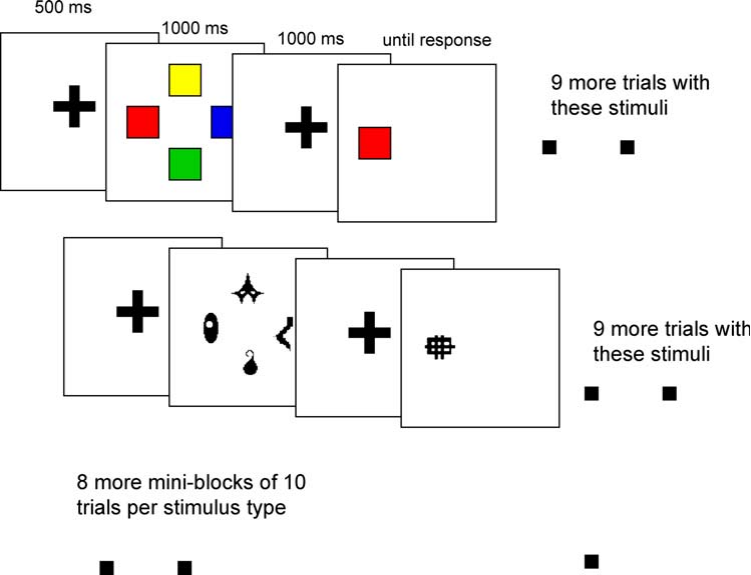
The design of one block of Experiment 2a. Experiment 2b was created by randomizing the trial order for 3 of the 6 blocks.

Each participant completed 600 trials, broken into blocks of 100. Each block consisted of 10 mini-blocks of 10 trials each. There was no pause between mini-blocks. The same stimulus set was used throughout a mini-block. No stimuli were used on more than one mini-block per block. The order of the mini-blocks was randomized on each block. The design of one block of Experiment 2a is given in Figure 2.

Note that, in contrast to Experiment 1, no-match test items were not explicitly controlled to have appeared on any particular previous trial. This was done in order to reflect a typical visual WM experiment. On trial 1 of a mini-block, a no-match test item was always novel, at least within that particular 100-trial block. On trial 10, a no-match test item was 89% likely to have appeared on trials 8 or 9, and 96% likely to have appeared within the last three trials. Practice, feedback and articulatory suppression were as in Experiment 1.

#### Experiment 2b

The procedure for Experiment 2b was a modification of Experiment 2a, where the trial order was randomized for three of the six blocks, thus disrupting the “mini-block” design for those three blocks, leaving the other three unchanged. Thus, the *blocked* condition was identical to Experiment 2a and should exhibit increasing PI across each “mini-block”. The *randomized* condition should exhibit minimal PI. Otherwise, the method of the two experiments was identical.

### Experiment 3: Method

#### Participants

Twenty participants completed Experiment 3a. Ten participants completed Experiment 3b.

#### Materials

All stimuli were from the non-nameable novel shapes group. In Experiment 3a, ten different colors were used, creating ten monochromatic stimulus groups. In Experiment 3b, as in the previous experiments, all six shapes were gray.

#### Experiment 3a

The procedure was identical to Experiment 2a in all ways except that the ten stimulus groups used ten differently-colored versions of the non-nameable novel shapes. Each stimulus group was monochromatic. Thus, the stimuli on the first trial of each block were novel by virtue of having a new color relative to the previous trial (the *high-novelty* condition). On the 10^th^ trial, the stimuli are far less novel (the *low-novelty* condition).

#### Experiment 3b

The procedure was identical to Experiment 3a with two exceptions. The color of the stimuli was grey and did not change between mini-blocks. Instead, participants were given a short pause between each mini-block and told to press the spacebar when they were ready to continue, effectively creating 60 identical blocks of 10 trials each. Thus, on trial 1 of each block, participants were relatively refreshed (the *refreshed condition*), while by trial 10 they are likely to be more fatigued (the *fatigued condition*).

### Experiment 4: Method

#### Participants

Participants were anonymous volunteers recruited via the Internet (www.vacognition.wjh.harvard.edu). Consent followed the same procedure as in the above experiments, except that the participants clicked a link to indicate consent. A total of 3,185 first-time participants 18–40 years old who reported normal or corrected-to-normal vision completed the experiment.

#### Equipment

The experiment was programmed in Flash MX. The program downloaded in its entirety before running. Data was recorded in a MySQL database via PHP. Participants were asked to be in a quiet environment where they would not be distracted for the duration of the experiment (about 5 minutes).

#### Materials

Stimuli were six non-nameable novel objects used in the previous experiments and shown at the bottom of Appendix S1. Shapes ranged from approximately 60×60 pixels in diameter and were displayed on a neutral gray background 500×400 pixels in size.

#### Procedure

Each trial began with 500 ms of fixation. Four stimuli were displayed around an imaginary circle 75 pixels in radius for 1000 ms, followed by a further 1000 ms of fixation. A test item appeared in one of the four previously-occupied locations. Participants used their mouse to click a button to indicate whether the test item matched or did not match the memory item that was in that location.

The experiment, which consisted of 40 trials in a single block, was preceded by 10 trials of practice (which participants could repeat if desired). Practice stimuli (Greebles, used with permission from Michael J. Tarr, http://www.tarrlab.org) were highly dissimilar from the experimental stimuli. The practice stimuli were also more challenging in order to encourage the participants to pay attention. Participants could repeat the practice session, but most did not. Participants received feedback only on incorrect responses. Articulatory suppression was induced by asking participants to repeat a one-syllable word out loud during the entire block. An archive of the experiment is maintained at the website (http://www.coglanglab.org/VSTMTime/).

## Results

### Experiment 1: Results

**Figure 3:**
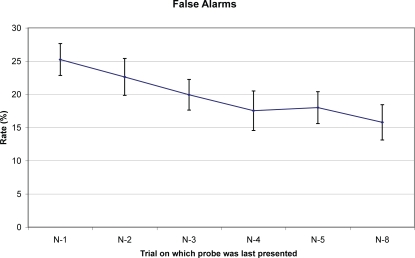
Experiment 1: False alarm rates (with standard errors) across as a function of probe's last presentation.

Participants correctly accepted the test item in match trials 78% of the time (SD = 13%). The false alarm rate in the six no-match conditions is given in Figure 3. The six conditions differed significantly (F(1,19) = 9.22, p<.001). Participants were significantly more accurate in the N-8 (∼baseline) condition than in the N-1 (t(19) = 5.57, p<.001), N-2 (t(19) = 5.44, p<.001), or N-3 conditions (t(19) = 3.07, p = .006). Participants were non-significantly more accurate in the N-4 (t(19)<1) and N-5 (t(19) = 1.66, p = .11) conditions.

Thus, Experiment 1 does show that item-specific PI requires at least several trials to decay. This suggests that in a typical experiment with many trials and small numbers of stimuli, it is quite likely that all non-match probes are “recent probes,” and thus subject to item-specific PI. This suggests that item-specific PI could in fact play an important role in determining estimates of visual WM capacity.

On the other hand, the effects shown in this experiment are not large and do not suggest that eliminating PI would dramatically increase measured visual WM capacity. That said, all our conditions are subject to considerable item-nonspecific PI. In Experiment 2, we get a cleaner measure of visual WM capacity absent PI.

### Experiment 2: Results

#### Experiment 2a

**Figure 4:**
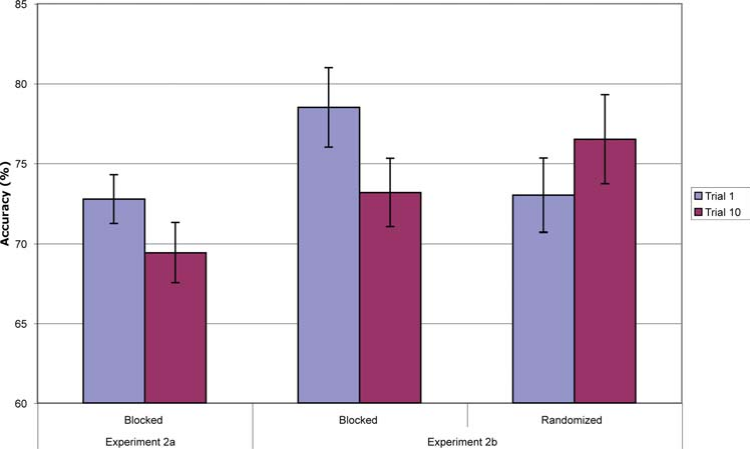
Accuracy (with standard errors) for the first trial (low proactive interference) and last trial (high proactive interference) of each mini-block in Experiments 2a and 2b (note that the in Experiment 2b, the first and tenth trials are both low proactive interference trials).

Mean accuracy across the 10 mini-block trials is given in Figure 4. Analyses on accuracy, d-prime and a-prime all gave similar results; only accuracy analyses are reported. A planned paired t-test between the first trial (low-PI) and the tenth (high-PI) was significant (t(19) = 2.20, p = .04) in the predicted direction.

Capacity of visual short-term memory can be estimated using Cowan's K: (hit rate+correct rejection rate–1) * number of items to be remembered [13], [32]. Using this formula, measured capacity decreased from 1.8 (SD = 0.5) on the first trial of each mini-block to 1.5 (SD = 0.7) on the tenth trial. This difference was significant (t(19) = 2.20, p = .04). This reduction was due entirely to an increase in false alarms (from 22.9% to 29.5%); the hit rate stayed constant (68.2% vs. 68.1%), a finding consistent with the effects of PI.

#### Experiment 2b

For the purposes of analysis, the *randomized* blocks were also divided into 10-trial mini-blocks. Mean accuracy is given in Figure 4. In the *blocked* condition, accuracy on trial 1 (the low-PI condition) was significantly higher than on trial 10 (the high-PI condition; (t(19) = 2.44, p = .02), replicating Experiment 2a. As in Experiment 2a, this was due to an increase in false alarms (19.2% to 27.7%) rather than a decrease in hits (75.9% to 76.0%). In the *randomized* condition, accuracy on trial one was non-significantly lower than on trial ten (t(19) = 1.29, p = .21). A 2×2 repeated-measures ANOVA on *blocked*/*randomized* vs. *low-interference/high-interference* revealed a significant interaction (F(1, 19) = 6.53, p = .02).

#### Combined

It is unlikely that PI causes performance to fall continuously, but from the data so far reported, it is not clear if and where performance asymptotes as PI builds.

**Figure 5:**
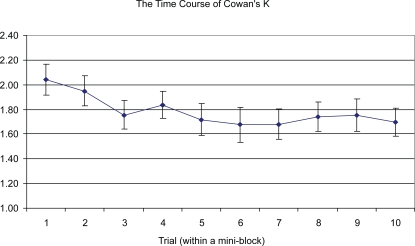
The decline in estimated WM capacity in terms of Cowan's K (with standard errors) as a function of trial number within the “mini-blocks” of Experiment 2a and the blocked condition of Experiment 2b. N = 41.

Figure 5 plots average performance across the ten trials of the “mini-blocks,” averaging over Experiment 2a and the *blocked* condition of Experiment 2b. Results for the experiments individually were roughly similar. From the plot, it appears that the decline in performance levels off within no more than 5 or 6 trials. This corresponds well with the results of Experiment 1, in which item-specific PI appeared to decay entirely within 4 or 5 trials.

### Experiment 3: Results

**Figure 6:**
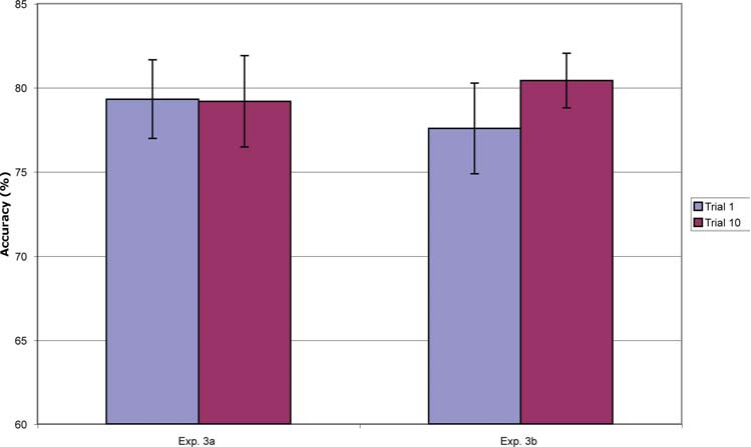
Mean accuracy (with standard errors) for Experiments 3a and 3b, trials 1 and 10 of each mini-block.

Results from Experiment 3a are shown in Figure 6. Accuracy in the first trial (*high-novelty condition*) and last trial (*low-novelty condition*) was nearly identical. The difference was not significant (t(19) = .09, p = .93).

Results from Experiment 3b are shown in Figure 6. Experiment 3b was stopped after testing ten participants because it was clear that the effect was if anything in the wrong direction for the *attentional hypothesis*. Accuracy in the first trial (*refreshed condition*) was somewhat lower than in the last trial (*fatigued condition*), a difference that was not significant (t(19) = 1.14, p = .28)–opposite from the predictions of the *attentional hypothesis*.

Experiments 3a and 3b thus fail to confirm the *attentional hypothesis*. That is, they suggest that the results of Experiments 2a and 2b were not due merely to increased novelty at the beginning of each mini-block, leading to participants paying more attention on those trials. In Experiment 3a, the monotonous onward march of trials within 100-trial blocks was broken up by switching the color of the stimuli. In Experiment 3b, a short rest was inserted between every 10 trials of the 100-trial blocks. Although either or both of these manipulations should have recaptured the participant's attention, if in fact attention was wandering, neither manipulation was sufficient to increase accuracy on the first trial of each mini-block. While it may be that only switching the stimulus set is sufficient to recapture attention enough to cause a boost in accuracy, it is not clear how that would be de-confounded from PI or whether that is in fact different from PI.

### Experiment 4: Results

**Figure 7:**
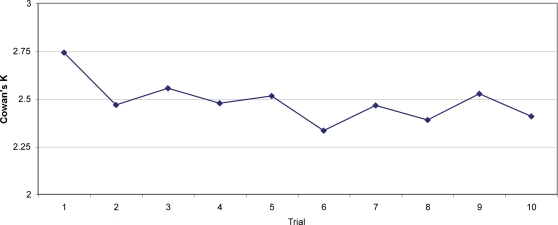
Cowan's K for the first 10 trials of Experiment 4 (error bars are not displayed because Cowan's K cannot be calculated for individual participants, who either got each item correct or incorrect).

In order to facilitate comparison with Experiment 2, Cowan's K for the first 10 trials only are shown in Figure 7 (the results from the subsequent trials are interesting but outside the scope of the present paper; they will be presented in a future paper currently in preparation). The pattern of results for accuracy is qualitatively similar.

Cowan's K dropped by 12.1% from the first trial to the 10^th^, a smaller change than that in Experiment 2, but one of a roughly similar degree. This difference is significant in a McNemar test on accuracy (χ^2^ = 18.45, p<.001; no tests are possible on Cowan's K, because here it cannot be calculated individually for each participant). This confirms that although PI has a statistically significant impact on measured visual WM capacity, it does not affect capacity enough to explain away the differences between measured visual and verbal WM capacity.

The fact that the results of this experiment agree so well with those of the in-lab experiment is further evidence that Web-based experiments can be reliable sources of data.

## Discussion

There are a few details from the above results that deserve some discussion before turning to the larger questions in this study.

All the Cowan's Ks reported above are well below the standardly-claimed 3–4 object capacity of visual WM. However, this is not without precedent, having been reported for many types of stimuli in the past [2], [5]. One explanation is that capacity may be less for more complex objects [2], though recent evidence also suggests that the ease of discriminating no-match probes from the memory items also plays a role [3]. This latter point may suggest that probe-based WM tests are not so much measures of the number of items that can be stored, but of the number of items that can be stored with enough fidelity to discriminate them from the probe. This is either appropriate or inappropriate depending on how it compares to how WM is actually used “in the wild,” an issue which may not have received enough attention. It should be noted that verbal WM capacity outstrips visual WM capacity in probe-based tasks also [43], so this caveat about discriminability is somewhat tangential to my present purposes.

In Experiment 2b, it is interesting to note that performance on the low-PI *randomized* blocks was intermediate between performance on the first and last trials of each mini-block on the *blocked* blocks. This may be due to the fact that average PI on the first trial of a *blocked* mini-block (in which that stimuli type had not been viewed on average for 50 trials) was lower than on any given trial of the *randomized* blocks (in which, due to how the randomization was done, stimuli of that type had probably been seen more recently). Thus, looking at the first trial that uses a particular set of stimuli may give the cleanest measure of visual WM capacity.

It is worth comparing these results to two previous studies that used a similar design [28], [29]. The authors presented participants with a WM task similar to those used here. As in Experiment 2, there were short blocks of trials in which the same stimulus set was used. From block to block, the stimulus set varied. Unlike the present study, different stimulus sets included visual objects, locations and also visually-presented verbal material (e.g., names of flowers). Also, the authors reported reaction times (RTs), not accuracy. There were several different experiments, which differed in terms of the length of the blocks and whether there were breaks between blocks.

In the several different experiments, the authors found consistent results: RTs were fastest on the first trial of the block, increased through the 3^rd^ or 5^th^ trial, and then quickly fell again to a very noisy intermediate level. This contrasts with the accuracy data here, where performance did not recover, though this may be a difference between accuracy and reaction time. Although it is not clear in all their experiments how many trials were used, where the number of trials is reported, it is considerably fewer than the 600 trials used here. So it is also possible that their data was simply less robust. Furthermore, since they did not break down their data into verbal stimuli and visual stimuli, it is not known whether their effect at the later trials was due differentially to one or the other. However, if RT and accuracy do in fact dissociate in these experiments, that may be worth further exploration.

### General Discussion

One of the most salient results from research in visual WM is its severely limited capacity [2], [33], [44]. It remains an open question the degree to which these measured limitations are experimental artifact. Certainly, variation in experimental design can dramatically affect measured capacity [3]. Given that typical visual WM capacity studies invite a great deal of proactive interference (PI), one possibility was that PI was artificially depressing capacity measures.

The results of the present study suggest that while PI certainly plays a role in visual WM performance, it does not dramatically change WM capacity estimates–at least for these sorts of stimuli and in these sorts of tasks. In Experiment 1, item-specific visual PI was estimated to reach asymptote within 4–5 trials. In Experiment 2, the combined effect of item-specific visual PI and item-nonspecific visual PI on measured visual WM capacity was estimated at a 17% reduction in capacity. A similar number (12%) was found in Experiment 4, which used a cleaner low-PI baseline. Experiment 3 eliminated some methodological concerns about Experiments 2 and 4.

Thus, I cannot reject the twin hypotheses that (1) visual WM's severely limited capacity is due to experimental artifact, and (2) visual WM's limited capacity in relation to verbal WM is experimental artifact.

There are many other ways in which experimental artifact could be playing a role, and those remain to be tested. For instance, Awh and colleagues have demonstrated that for typical visual WM tasks (like those employed here), the degree of discriminability of the stimuli can have a massive effect on performance, which can serve to dramatically decrease measured visual WM capacity [3]. This problem does not arise in typical full-report verbal WM tasks, which do not use probes and thus do not require similar discrimination.

At least two directions of research could address this potential confound. One would be to systematically compare verbal and visual WM capacity using the probe method. In fact, researchers since Sternberg have probed verbal WM with this task [43]. In Sternberg's original paper, he found ceiling accuracy for the probe task even when six words were being maintained in WM–well beyond any reports for visual WM capacity. (I am assuming that, although the digits were presented visually, they were maintained in verbal memory. This may require testing.) However, it may be necessary to carefully equate discriminability for both the visual and the verbal stimuli in order to make a direct comparison.

The other direction would be to test visual WM with the full-report paradigm. This is very similar to what Zhang and Simon pioneered [16]. However, it is not clear whether this paradigm can be readily extended beyond Chinese characters. Even within Chinese characters, it will be a non-trivial task to control the stimuli on every possible relevant factor (visual complexity, discriminability, familiarity, ease-of-naming, etc.).

There are other potential methodological confounds as well. For instance, in verbal WM tasks, sensory information (e.g., pitch, tenor, prosody) can typically be discarded as irrelevant. However, visual WM tasks often focus on specifically sensory aspects of the stimuli (e.g., size, color; I thank Johan Lauwereyns for this suggestion).

Should all methodological issues be addressed and the differences between visual and verbal WM capacity remain, the answer to this puzzle must be found in the nature of the human memory system itself. There are several intriguing possibilities. WM capacity is greater for words than non-words [45], and relatively little familiarization is required to increase capacity for non-words [31]. In contrast, long-term memory traces have little to no impact on visual WM [1], [13], [14], [15]. Similarly, while explicit strategies have been known to improve verbal WM capacity by an order of magnitude [9], [10], there are no such reports for visual WM. However, the literature on the role of long-term memory or explicit strategies in visual WM capacity is limited, and it does not appear any systematic comparison with verbal WM has been attempted. Moreover, even if visual and verbal WM capacity differences can be reduced to differences in strategy use or the impact of long-term memory, that will only raise the question of why visual WM lacks these features.

Thus, more research on these questions is needed.

One purpose of this study was to investigate the reliability of the strangely small visual WM capacity estimates reported in the literature. Another was to further investigate the nature of proactive interference (PI) in visual WM. PI has only been very recently described in visual WM [17], [28], [34], [35], and thus much about its nature is not understood, including how it compares with verbal PI. One recent study found inconsistent results across stimulus types and modalities, both behaviorally and with neuroimaging [28]. Moreover, Mecklinger, Weber, Gunter and Engle [34] found that while participants with large verbal WM capacity do not show significant verbal PI (as predicted, since the two are inversely correlated), they do show significant PI for abstract objects, suggesting that resistance to PI for verbal stimuli may not perfectly predict resistance to PI for visual stimuli.

Thus, the present study contributes to the understanding of visual WM PI in the following ways. First, it replicates previous claims of its existence using a variety of visual stimuli (Experiments 2). Second, I was able to measure PI on the first several trials using novel stimuli (Experiment 4), something which has been done for verbal materials [24], [26], [46] but was not previously accomplished for visual materials due to the large samples required. This serves as continuing evidence that Web-based experimentation is a powerful technique for testing hypotheses difficult to test in a traditional lab-based setting.

I also showed that, using the recent probes paradigm, one can find evidence of item-specific visual PI across at least 3 trials and a dozen seconds (in fact, in another experiment not presented here, I found that visual PI decays as a function of the number of intervening stimuli, not the passage of time).

Nearly all other investigations of item-specific PI consider only probes most recently seen on trial N-1. The only other study comparable to Experiment 1 that I know of is Monsell [36]. In that study of verbal WM, reaction times were longer and false alarms more frequent for probes most recently presented on trial N-2 than N-4 and N-4 than N-6. This was significant in an omnibus F test, but no pair-wise comparisons were reported, limiting any conclusions. However, these results are in the same ballpark as what I found for visual PI.

Moreover, Experiments 2 and 4 demonstrated that the cumulative effects of item-specific and item-nonspecific PI reach asymptote by the third to fifth trial, even under conditions designed to promote PI. Experiments 3 ruled out the possibility that these effects are due to observers paying more attention to sudden changes in stimuli, a plausible counter-explanation to PI which interestingly does not appear to have been tested previously in the verbal or visual domains. A couple early studies appear to suggest a similar asymptote for verbal PI as well [24], [46].

Thus, visual and verbal PI–particularly item-specific PI–seem similar in some respects, suggesting potentially a common mechanism or at least two very similar mechanisms. However, in an additional experiment, I found that increasing the retention interval from 250 ms to 4300 ms did not affect PI, which contrasts with reports that retention interval correlates positively with verbal PI [24], [46]. However, that study found this effect to be much reduced for short retention intervals such as were used in the present experiments. Thus, while this indicates a potential difference between visual and verbal PI, it is far from conclusive.

The similarity and differences between visual and verbal PI may help clarify models of memory. Here, I consider these results in terms of two very recent accounts of PI.

Makovski & Jiang [17] seem to assume a multi-store model with separate long-term and short-term components when they suggest that item-specific PI is caused by inefficient removal of unneeded items from the WM buffer [47]. Though a multi-store model could accommodate identical behavioral signatures of visual and verbal PI, such commonalities across the different WM buffers does not seem necessary and would need to be explicated.

In contrast, Jonides and colleagues [48] provide some discussion of PI in terms of a single-store model, in which WM is a set of processes that activate long-term memory representations more or less *in situ* (i.e., no separate buffer is involved). In their account, PI operates through two possibly distinct mechanisms: (1) recent exposure to similar items may increase the noise that affects the fidelity of the representation of the current to-be-remembered item causing memory decay, and (2) retrieval of an item from WM requires reinstantiating its active firing pattern, which would be subject to interference from similarly encoded patterns. Thus, the Jonides et al. account more parsimoniously deals with similarities between visual and verbal PI, since their account pertains to the mechanisms of memory quite independent of modality.

A number of questions about visual PI remain open for the future. Despite the importance of PI and WM, PI in visual WM has received relatively little attention. In verbal WM, an individual's susceptibility to PI has a strong inverse relationship with their verbal WM capacity [27]. However, it is not known whether the same relationship is true for visual WM. Similarly, verbal PI susceptibility appears to be a strong negative predictor of general intelligence [49], [50]. It is not know whether the same is true for visual PI. Similarly important, recent structural equation modeling work suggested that executive function may decompose into two components: shifting/updating and resistance to PI [51], but that study relied exclusively on tests of verbal PI.

### Conclusion

Visual working memory capacity appears to be extremely limited, though a number of potential confounds have not been eliminated. One potential confound, an out-sized effect of proactive interference, has been eliminated. However, more work is left to be done.

## Supporting Information

Appendix S1 Appendix: Stimuli used in experiments: colors, cubes, faces, fribbles, houses, orientations, polygons, Sanskrit, rotated numbers, novel shapes.
(5.49 MB TIF)
