# Supplementary figures and images for: Visual Working Memory Capacity and Proactive Interference
Source: PLoS One. 2008 Jul 23;3(7):e2716. doi: 10.1371/journal.pone.0002716 (PMC2447156; doi:10.1371/journal.pone.0002716)

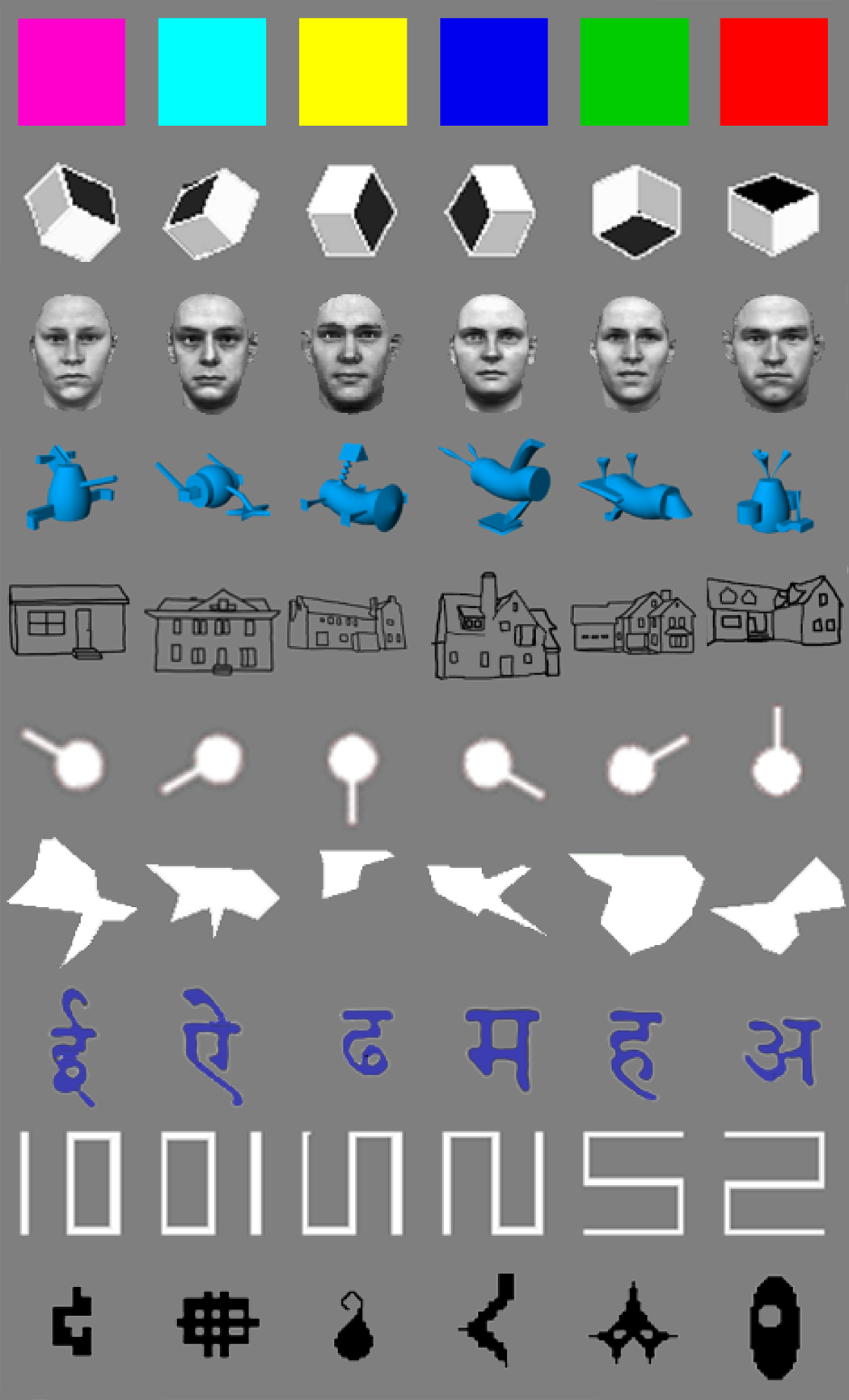

Supplement: Appendix S1 — Appendix: Stimuli used in experiments: colors, cubes, faces, fribbles, houses, orientations, polygons, Sanskrit, rotated numbers, novel shapes. (5.49 MB TIF) [file pone.0002716.s001.tif]
